# Acute Cardiotoxicity Evaluation of the Marine Biotoxins OA, DTX-1 and YTX

**DOI:** 10.3390/toxins7041030

**Published:** 2015-03-27

**Authors:** Sara F. Ferreiro, Cristina Carrera, Natalia Vilariño, M. Carmen Louzao, Germán Santamarina, Antonio G. Cantalapiedra, Luis M. Botana

**Affiliations:** 1 Departamento de Farmacología, Facultad de Veterinaria, Universidad de Santiago de Compostela, 27002 Lugo, Spain; E-Mails: sara.fernandez.ferreiro@usc.es (S.F.F.); cristina.carrera@usc.es (C.C.); mcarmen.louzao@usc.es (M.C.L.); 2 Hospital Veterinario Universitario Rof Codina, Facultad de Veterinaria, Universidad de Santiago de Compostela, 27002 Lugo, Spain; E-Mails: german.santamarina@usc.es (G.S.); antonio.cantalapiedra@usc.es (A.G.C.); 3 Departamento de Ciencias Clínicas Veterinarias, Facultad de Veterinaria, Universidad de Santiago de Compostela, 27002 Lugo, Spain

**Keywords:** okadaic acid, dynophysistoxins, yessotoxin, cardiotoxicity, hERG, ECG, cardiac biomarkers

## Abstract

Phycotoxins are marine toxins produced by phytoplankton that can get accumulated in filter feeding shellfish. Human intoxication episodes occur due to contaminated seafood consumption. Okadaic acid (OA) and dynophysistoxins (DTXs) are phycotoxins responsible for a severe gastrointestinal syndrome called diarrheic shellfish poisoning (DSP). Yessotoxins (YTXs) are marine toxins initially included in the DSP class but currently classified as a separated group. Food safety authorities from several countries have regulated the content of DSPs and YTXs in shellfish to protect human health. In mice, OA and YTX have been associated with ultrastructural heart damage *in vivo*. Therefore, this study explored the potential of OA, DTX-1 and YTX to cause acute heart toxicity. Cardiotoxicity was evaluated *in vitro* by measuring hERG (human èter-a-go-go gene) channel activity and *in vivo* using electrocardiogram (ECG) recordings and cardiac damage biomarkers. The results demonstrated that these toxins do not exert acute effects on hERG channel activity. Additionally, *in vivo* experiments showed that these compounds do not alter cardiac biomarkers and ECG in rats acutely. Despite the ultrastructural damage to the heart reported for these toxins, no acute alterations of heart function have been detected *in vivo*, suggesting a functional compensation in the short term.

## 1. Introduction

Marine algal blooms are natural phenomena caused by the overgrowth of marine phytoplankton. Presently, their appearance seems to have increased in frequency and severity, suggesting a public health problem of worldwide distribution. Some phytoplankton species present in these blooms produce phycotoxins that get accumulated in edible tissues of filter feeding shellfish through the marine food webs. Human intoxication episodes occur when contaminated seafood is consumed.

Diarrhetic shellfish poisoning (DSP) is the toxic syndrome caused by the ingestion of shellfish contaminated with okadaic acid (OA) or its analogues, the dynophysistoxins (DTXs). OA and DTXs are marine lipophilic phycotoxins produced by dinoflagellates of the genera *Prorocentrum* and *Dinophysis*. DSP’s first episode was documented in Japan in the 1970s and since then, many episodes have been reported worldwide [1]. DSP is a severe gastrointestinal intoxication, of which the main symptoms are diarrhea, nausea, vomiting and abdominal cramps. Until now, no deaths have been related to acute or chronic toxicity of these toxins [2]. In order to protect human consumers, the presence of DSPs in seafood is regulated in many countries [3]. The mechanism of action of OA and DTXs is most likely related to the potent inhibition of serine/threonine protein phosphatases [4,5], which leads to hyperphosphorylation of cell proteins and dis-regulation of many cellular processes. Several toxicological studies with these compounds have described many effects at different cellular, molecular and genetic levels. In fact, they have been reported to cause cytotoxicity, neurotoxicity, immunotoxicity and embryotoxicity, as well as genotoxicity, tumor promotion and carcinogenicity [2].

Yessotoxins (YTXs) are polycyclic ether compounds produced by the phytoplanktonic dinoflagellates *Protoceratium reticulatum*, *Lingulodinium polyedrum* and *Gonyaulux spinifera.* YTXs were initially included in the DSP toxin class because they were detected simultaneously with OA and DTXs; however, nowadays they are classified and regulated separately owing to their different chemical structure, the lack of phosphatase inhibition activity and the absence of gastrointestinal toxicity [3]. Actually, and even though these toxins are also distributed worldwide, no human intoxication episodes have been related to the presence of yessotoxins in seafood [6]. *In vivo* toxicological studies in mice showed that yessotoxin causes alterations mainly in heart muscle [7,8,9,10]. On the other hand, *in vitro* data indicate that YTX induces apoptosis in many different cell lines, and it has been implicated in alterations of calcium movement [11], cyclic nucleotides and phosphodiesterases [12], or E-cadherin pathway and cytoskeleton [6,13]. Although the complete mechanism of action is not yet elucidated, the interaction of YTX with PDE4 is linked to the translocation to membrane and nucleus of the AKAP 149-PKA-PDE4A complex [14,15], being as well this effect linked to mTOR in apoptosis or autophagia [16].

In spite of the reports of ultrastructural alterations of cardiac muscle after oral or intraperitoneal administrations of yessotoxin and okadaic acid in mice [7,8,9,10,17,18], the functional implications of these effects have not been studied. The evident mitochondrial damage in one of the tissues with the highest demands of energy suggests potential cardiotoxicity [7]. In fact, the recommendations of the last EFSA report on YTXs, elaborated by a panel of experts on this field, include the study of the toxicological significance of these ultrastructural changes described in the heart [19].

Guidelines for the evaluation of a compound potential cardiotoxicity can be found in the recommendations of the EMA [20] and in several articles [21,22], entailing both *in vivo* and *in vitro* experiments. The evaluation of the effect on hERG (human èter-a-go-go gene) channel function by patch clamp is the acute *in vitro* method of choice to assess cardiac safety in drug development [23]. HERG encodes the channel responsible for a critical current in cardiac action potential (AP) repolarization, the rapid delayed rectifier K^+^ current (I_Kr_) [24]. Alterations of HERG channel currents have been related to the appearance of arrhythmias, specifically a type of fatal arrhythmia known as “Torsades de Pointes” (TdP) [25]. Additionally, some drugs can alter hERG by disruption of channel trafficking, but this is considered a chronic effect that usually takes hours or even days to occur [26]. Even though all cardiac channels contribute to the coordinated electrical activity of the heart, the implications on heart conductivity of hERG dysfunction have made hERG blockage evaluation essential to estimate potential cardiotoxicity. To evaluate *in vivo* heart toxicity, heart function and structural damage are considered. For functional alterations, electrocardiography is the technique of choice [21,22,27]. An electrocardiogram (ECG) represents the changes of electrical charge of the heart chambers for every beat; therefore it gives information about the overall electrical activity of the heart. The ECG may be altered by changes in biochemical and metabolic processes, by modifications of cardiac channels and cardiomyocyte membrane properties and by any structural injury that affect impulse generation and propagation [27]. In relation to structural damage, plasma cardiotoxicity biomarkers have also been recently included among the *in vivo* experiments for assessing cardiac toxicity [28]. Cardiac troponins I (cTnI) and T (cTnT) and the brain natriuretic peptide (BNP) are among the more accepted cardiac biomarkers nowadays [29,30].

Therefore, the aim of this work was to evaluate OA, DTX-1 and YTX acute cardiotoxicity using *in vivo* and *in vitro* methods.

## 2. Results and Discussion

### 2.1. OA and YTX Effects on hERG Channel Activity

**Figure 1:**
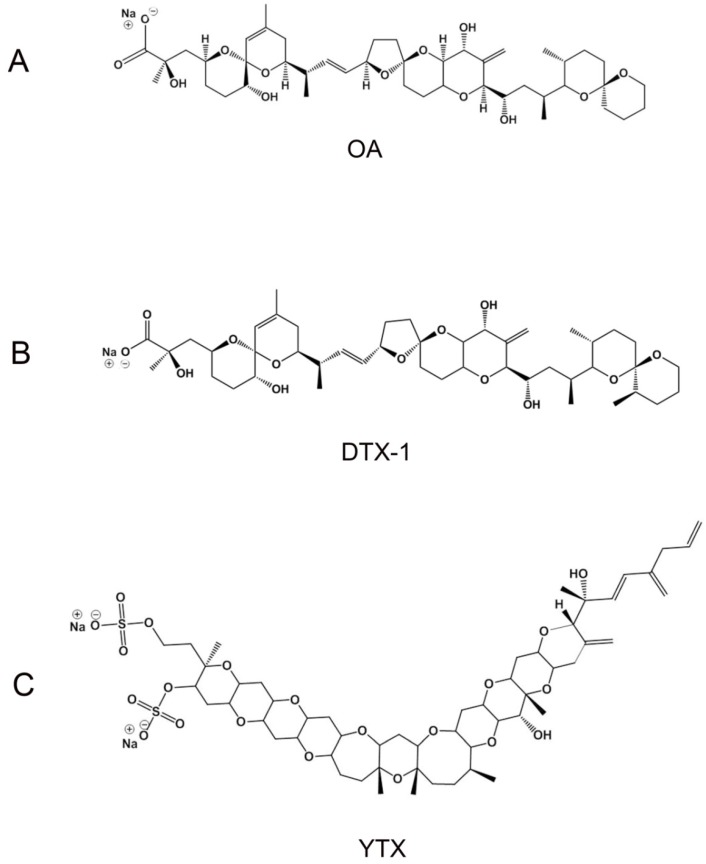
(**A**) Okadaic acid (OA); (**B**) dynophysistoxin-1 (DTX-1); (**C**) yessotoxin (YTX) chemical structures.

**Figure 2:**
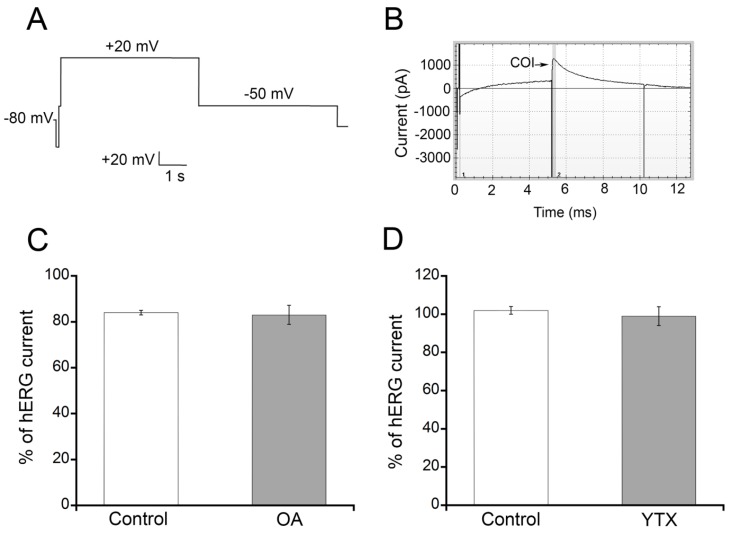
**Effects of OA, DTX-1 and YTX on human èter-a-go-go gene (hERG) channel activity.** Automated patch clamp experiments were performed using a CHO cell line stably expressing hERG. (**A**) Voltage clamp protocol for the activation of hERG; (**B**) Representative current trace obtained after YTX addition. Currents were monitored for 5 min after toxin addition. COI: current of interest; (**C**) No effect of OA on hERG currents. Current magnitude is expressed as percentage of pre-treatment current that remained after 5 min of exposure to 10 µM OA or carrier alone (mean ± SEM; *n* = 4); (**D**) No effect of YTX on hERG currents. Current magnitude is expressed as percentage of pre-treatment current that remained after 5 min of exposure to 10 µM YTX or carrier (mean ± SEM; *n* = 4).

The ability to block hERG channel currents is one of the required tests for preliminary evaluation of cardiotoxicity in drug development. Therefore, the effects of OA and YTX (Figure 1) on hERG activity were explored using automated patch clamp for the measurement of hERG currents in a CHO cell line stably expressing this channel. HERG channels were activated with the voltage protocol shown in Figure 2A. After stabilization of hERG currents, the cells were exposed to 10 µM OA or YTX, or an equivalent concentration of DMSO (carrier) for 5 min. The Ionflux system warrants an immediate change of extracellular solution and therefore no time is needed to allow for drug diffusion. For routine screening of hERG inhibiting drugs it is common to test concentrations in this range that will cause a clear and fast inhibition if the drug has high to medium blocking potency. A representative image of the current displayed by hERG CHO cells after YTX addition appears in Figure 2B. OA (Figure 2C, grey bar) and YTX (Figure 2D, grey bar) did not alter I_Kr_ amplitude after 5 minutes of exposure to a toxin concentration of 10 µM, when compared to the current in the same cells just before toxin addition. Similar results were obtained in cells exposed to carrier alone; no difference of I_Kr_ before and after addition of DMSO was observed (Figure 2C,D, white bars). DTX-1 (Figure 1) is an analog of OA and it has been described to have similar potency [1,31,32], thereby it was not tested in this assay to save the limited amount available for *in vivo* studies, which will provide information about potential cardiotoxicity generated by several possible mechanisms. Concentrations higher than 10 µM were not tested considering that compounds with an IC_50_ between 1 and 100 µM are classified as low potency hERG blockers and therefore would be pathologically irrelevant [33]. An underestimation of blocking potency due to technique limitations is not probable because the IC_50_ obtained for cisapride, a well-known high-potency hERG blocker, in our experimental conditions was 3 nM [34].

### 2.2. Effects of OA, DTX-1 and YTX on Rat ECG

**Figure 3:**
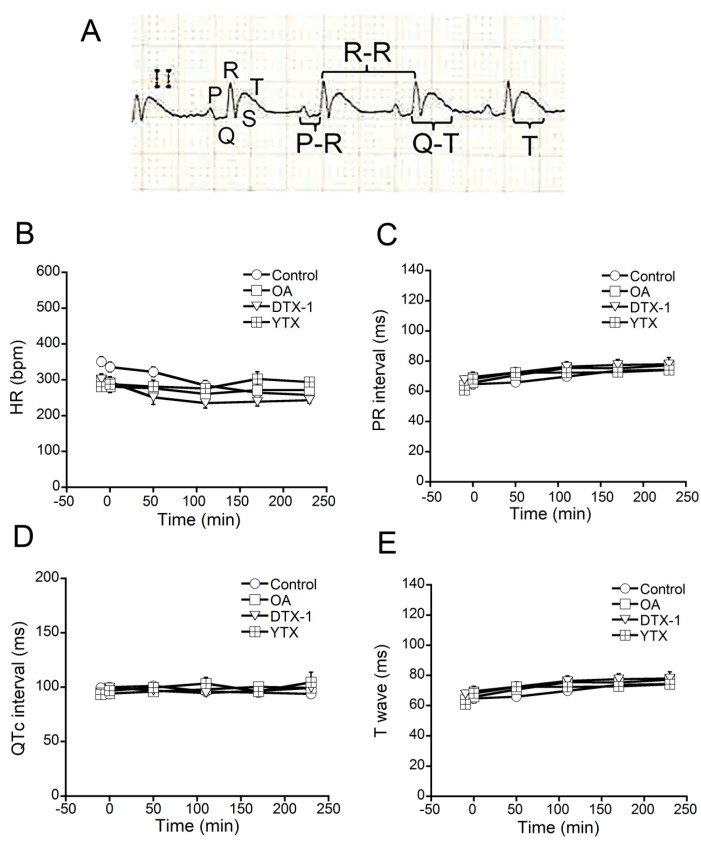
**Effects of OA, DTX-1 and YTX on rat electrocardiogram (ECG).** ECG parameters (HR, PR interval, QT_c_ interval and T wave) were analyzed before and at different times after intravenous administration of 20 µg/kg OA to 5 rats, 16 µg/kg DTX-1 to 5 rats and 10 µg/kg YTX to 7 rats. (**A**) Representative ECG recording at 50 mm/s. The landmarks and the measurements of PR interval, R-R interval, QT interval and T wave duration are indicated; (**B**) HR; (**C**) PR interval; (**D**) QT_c_ interval and (**E**) T wave were measured for all treated rats in ECG recordings at the following times: −10 (before toxin administration), 0 (toxin administration), 50, 110, 170 and 230 min. At every time point ECG was recorded for 10 min.

Cardiotoxicity may be caused by many mechanisms different than hERG blockage. Therefore, studies of potential heart toxicity were performed *in vivo*. The animal model selected for this study was the rat, due to practical feasibility reasons, because, owing to its size, rats provided an adequate balance between toxin expense, a correct manipulation during surgery and catheter placement for blood sample collections and reliable ECG results in our experimental conditions. Moreover, females were used due to the smaller size of adult individuals in relation to males, which allowed reducing the expense of toxin. The toxins were administered intravenously because this administration route is the fastest and requires lower toxin amounts. The effects of OA, DTX-1 and YTX on heart functionality were evaluated by electrocardiography. Several ECG parameters were analyzed before and at different times after intravenous administration of each toxin. Lead II and an ECG recording speed of 50 mm/s were used to measure HR (bpm), PR interval, QT interval and T wave durations (ms) (Figure 3A). ECG activity was recorded before, and immediately and every hour after toxin administration. Each recording had a minimal length of 10 min (continuous) and the total experiment duration was 4 h. For these experiments five rats were injected with 20 µg/kg OA, five rats with 16 µg/kg DTX-1 and seven rats with 10 µg/kg YTX. Nine control rats followed the same experimental procedure with the administration of carrier (DMSO in saline) in the absence of toxin. None of the ECG parameters evaluated were altered after the administration of OA, DTX-1, YTX or carrier (Figure 3B–E). Rodents have been widely used for cardiotoxicity evaluation and they share many similarities with humans in terms of pathophysiological changes or disease [27]; however, in humans hERG channel is an important player in heart action potential repolarization, and this is not the case in rodents. *In vitro* testing of hERG was included as a complement of cardiotoxicity evaluation due to this well-known difference. In addition, the macrolide antibiotic clarithromycin, which induces QT_c_ prolongation, was previously used as a positive control for QT_c_ interval prolongation in our experimental conditions; a dose of 2.2 mg/kg caused an increase of QT_c_ duration of 19% ± 3% [35,36].

Cardiac rhythm alterations were also analyzed. Type of arrhythmia, time of appearance from toxin administration, duration of arrhythmia sequences, total number of arrhythmia episodes, ECG recording duration and survival of the animal along the experiment are reported in Table 1. The rats administered with DTX-1 did not evidence any arrhythmia episode. However, 1/5 rat administered with OA, 2/7 rats administered with YTX and 2/9 rats administered with carrier showed the appearance of ventricular extrasystoles (VES). The total number of VES was 5 for OA-treated rats, 4 for YTX-treated rats and 3 for controls. Three YTX-treated rats died prematurely during the experiment as judged by apnea onset, and one of them had 3 VES before death occurred. One OA-treated rat died during the experiment but it did not show VES. None of DTX-1 and carrier-treated rats died before the end of the experiment. Therefore, it does not seem to be a correlation between death and arrhythmia occurrence in these experiments.

**Table 1:** Heart rhythm alterations in OA, DTX-1 and YTX-treated rats and controls.

Rat		Type	Time of Appearance	Duration (s)	Total N°	ECG Total Time (min)	Death before 240 min
**Control**	1				0	265	no
	2				0	265	no
	3				0	265	no
	4				0	265	no
	5				0	265	no
	6	VES	t68:45	3	2	265	no
	7	VES	t52:31		1	265	no
	8				0	265	no
	9				0	265	no
**OA**	10	0				265	no
	11	0				265	no
	12	VES	t110: 30	66	3	265	no
		VES	t174:45		1	265	no
		VES	t196:49		1	265	no
	13	0				130	yes
	14	0				265	no
**DTX-1**	15	0				265	no
	16	0				265	no
	17	0				265	no
	18	0				265	no
	19	0				265	no
**YTX**	20	0				160	yes
	21	0				265	no
	22	0				120	yes
	23	VES	t236:08	1	1	265	no
	24	VES	t77: 35	5	3	140	yes
	25	0				265	no
	26	0				265	no

VES: ventricular extrasystole.

The i.v. doses used in this study were 1/10 of mouse intraperitoneal LD_50_ for OA and YTX and 1/10 of mouse intraperitoneal MLD for DTX-1 (no lethality data available in rats) [32,37]. Actually, the i.v. dose of YTX is probably close to the LD_50_ by this route, since 3/7 rats died during the experiment. A fairly high dose was selected to be sure that if no effects were observed, there would be no need to repeat the experiment with a higher dose, since that kind of experimental design would require higher amounts of animals and toxin, and also to account for the possibly lower sensitivity of females to cardiovascular diseases and toxicity [38,39]. Thereby, if cardiotoxicity was an important component of acute YTX toxicity, some functional signs should have been observed at this dosing level. In the case of OA-treated rats, although this dose is clearly lower than the LD_50_, one death was recorded during the experiment. Therefore, this dose is enough to cause death of some individuals, but no cardiotoxicity signs were observed. DTX-1 toxic potency is considered similar to OA [1], although it might be higher by the oral route [40]. Thus, DTX-1 dosing levels would probably be sufficient to induce clinical signs if cardiotoxicity were an important component of DTX-1 toxicity. The lack of effects of YTX on the ECG is somehow surprising due to the abundant evidence of ultrastructural damage observed in cardiomyocytes of YTX-treated mice [7,8,9,10,17,18]. In fact, cardiomyocyte ultrastructural alterations have been described after intraperitoneal and oral administration of the toxin, even at doses that are far below the LD_50_ and at times as short as 1 h [8]. Furthermore, oral administrations of YTX at asymptomatic doses have demonstrated myocardial alterations at 24 h that persisted for at least 30 days [10,17]. The absence of effects on ECG recordings may be due to heart compensatory mechanisms to maintain functionality. Overall, OA, DTX-1 and YTX did not have *in vivo* functional effects on ECG parameters. Although some arrhythmia episodes were observed in toxin-treated rats, the frequency and number of animals affected were similar to controls, and therefore they would not be related to a toxin effect. Maintenance of functionality with this cardiomyocyte ultrastructural damage may not be feasible in the long term, considering that loss of cardiomyocyte function after injury is described as the principal etiology of heart failure [41], and therefore further studies are necessary to explore chronic cardiotoxic effects of these toxins.

### 2.3. Effects of OA, DTX-1 and YTX on the Levels of Cardiac Biomarkers

**Figure 4:**
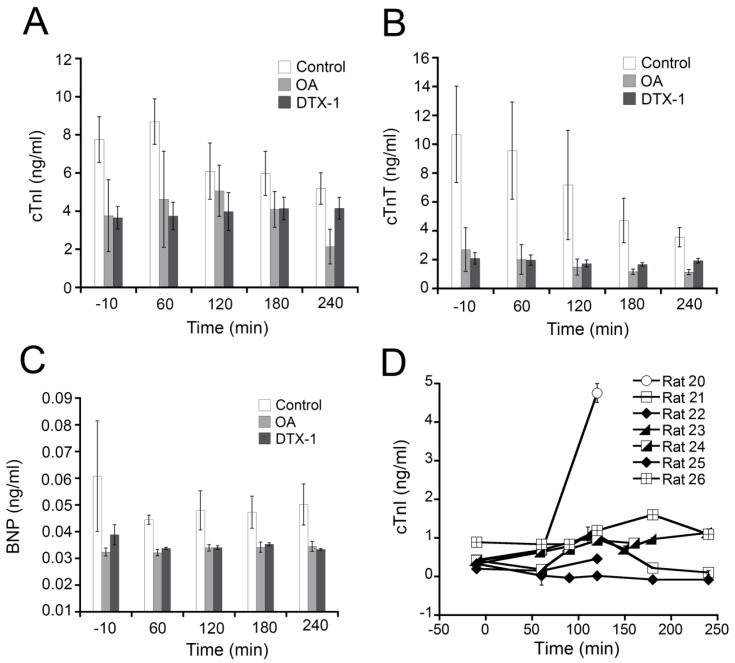
**Effects of OA, DTX-1 and YTX on the levels of cardiac biomarkers.** Blood samples were collected before and at different times (60, 120, 180 and 240 min) after i.v. administration of toxin or carrier. For OA- and DTX-1-treated rats, cTnI, cTnT and BNP were quantified in plasma samples. For YTX-treated rats cTnI levels were measured. (**A**) cTnI; (**B**) cTnT and (**C**) BNP plasma levels measured in OA- (light grey bars) and DTX-1-treated rats (dark grey bars) and control rats (white bars) using immunodetection with a xMap rat CVD panel from Millipore^®^; (**D**) cTnI plasma levels measured in seven YTX- treated rats using a specific ELISA kit. (Mean ± SEM).

Quantification of plasma cardiotoxicity biomarkers has also been recently included among the experiments for assessing *in vivo* structural heart damage. The levels of the cardiac biomarkers cTnI, cTnT and BNP were measured in rats treated with OA, DTX-1 or carrier. The quantification of these biomarkers was done in plasma samples collected during ECG experiments using panel 1 of the rat cardiovascular disease (CVD) kit from Millipore^®^. Plasma samples from YTX-treated rats were analyzed only for cTnI with a specific ELISA kit. Control samples from rats that received just the carrier were also included in the assays. Several plasma samples were collected for each rat along the experiment; one before toxin administration and the others every hour after toxin administration (see Methods section). Rats treated with OA or DTX-1 did not evidence any increase of plasmatic cTnI, cTnT or BNP during the experiment (Figure 4A–C, grey bars). The rats that received only the carrier (Figure 4A–C, white bars) showed similar levels of these biomarkers. The results for YTX-treated rats demonstrated no increase of cTnI during the first four hours following intravenous administration of the toxin in 6 rats (Figure 4D, rats 21–26), with the exception of one rat (Figure 4D, rat 20). Actually, no statistically significant differences were observed with regards to cardiac biomarkers in this study. In the case of rat 20, the animal entered apnea 140 min after toxin administration and cardiac activity continued for some time with increasing appearance of arrhythmias. Finally, the experiment was interrupted at time 160 min and a blood sample was collected that showed an increased concentration of cTnI. This increase that occurred after apnea onset was probably due to hypoxia and it should not be considered a direct effect of YTX. Isoproterenol, which is known to cause an increase of plasmatic cTnI [42], was used as positive control in these experimental conditions [36]. Two rats were injected with 4 mg/kg isoproterenol and after 2 h cTnI plasma levels were increased 3 times [36]. The detection of elevated cTnI plasma levels post-apnea and in isoproterenol-treated rats served as positive controls for the performance of immuno-detection techniques and experiment duration. Because the levels of cardiac cTnI and cTnT in plasma of rats treated with OA, DTX-1 and YTX were not altered, important myocardial injury after the acute administration of these toxins can be excluded [30]. Additionally, BNP levels, which are indicative of hemodynamic changes, ventricular damage or stress [43] were also not increased by OA and DTX-1.

### 2.4. Effects on Biochemistry Parameters

Heart dysfunction may be secondary to other organ damage. To provide a more complete pathophysiologic evaluation, several biochemical parameters were analyzed. A General Health Profile (GHP) prepacked panel was used to measure albumin (ALB), alkaline phosphatase (ALKP) alanine aminotransferase (ALT), blood urea nitrogen (BUN), calcium (Ca), cholesterol (CHOL), creatine kinase (CK), creatinine (CREA), globulin (GLOB), phosphorus (PHOS), and total protein (TP) in two plasma samples collected from each rat, one before toxin or carrier administration and the other after treatment, at the end of the experiment. The results are shown in Table 2. TP and ALB plasma levels were significantly lower after 4 h of experiment when compared to initial levels in all rats. GLOB levels were also decreased but only significantly in DMSO and DTX-1- treated rats. This reduction of TP, ALB and GLOB levels along the experiment was probably due to plasma dilution caused by fluid therapy. ALKP levels were also significantly reduced in all groups including controls. CHOL plasma levels were above normal values before DTX-1 administration and also before and after OA and YTX administrations. Additionally, CHOL levels were decreased at the end of the experiment in all rats; this lower values being statistically significant *versus* pre-treatment levels in YTX and DMSO-treated rats. Ca was slightly reduced at the end of the experiment in all groups. ALKP, CHOL or Ca decreases might also be explained by plasma dilution. In any case, the slight reductions observed in these parameters would not be clinically important in terms of organ functionality. CK levels were significantly elevated in OA and/or DMSO-treated rats; however they were always below normal values. ALT plasma levels seemed to elevate in DMSO, OA and YTX-treated rats, but only plasma concentrations of ALT in OA-treated rats reached values above the physiological range. ALT is the clinical chemistry gold standard of hepatotoxicity [44] and its increase would be in agreement with previously reported hepatic damage observed in rats after i.v. administration of OA [45]. BUN plasma levels were significantly increased and above the physiological range after OA and YTX treatment. Finally, CREA and PHOS plasma levels were within normal physiological values in all cases. CREA and BUN are the common biomarkers of nephrotoxicity and both are usually evaluated together when renal injury is diagnosed [46]. Our results did not show increased CREA levels, therefore BUN elevation might be indicative of incipient kidney damage but more parameters would be necessary to confirm a direct nephrotoxic action [46,47]. On the whole, these biochemical results showed only two alterations that could be related to the toxin treatment, the apparent ALT increase in OA-treated rats and the BUN increase in OA and YTX-treated rats. However none of these observations is definitely conclusive of liver or kidney damage.

## 3. Experimental Section

### 3.1. Chemicals and Solutions

OA (purity ≥ 98.9%), DTX-1 (purity ≥ 98%) and YTX (purity ≥ 97.9%) certified reference materials (CRMs) were supplied by Laboratorio CIFGA S.A. (Lugo, Spain). Nut Mix F-12 W/GLUTAMAX-I medium, fetal bovine serum (FBS), geneticin, trypsin/EDTA, CHO cell serum free media (CHO-SFM) and Dulbecco’s phosphate-buffered saline (DPBS) were purchased from Invitrogen^®^ (Eugene, OR, USA). Claritrhomycin (Klacid IV) was from Abbott Laboratories (Abbott Park, IL, USA). Isoproterenol (Aleudrina) was from Laboratorio Reig Jofre (Barcelona, Spain). Trypan blue solution, dimethyl sulfoxide (DMSO) and cisapride monohydrate were from Sigma-Aldrich Química S.A. (St. Louis, MO, USA). Detachin^TM^ was purchased from Labclinics (Barcelona, Spain). IonFlux extracellular solution (EC, 145 NaCl, 4 KCl, 1 MgCl_2_, 2 CaCl_2_, 10 HEPES, 10 Glucose (mM)), IonFlux intracellular solution (IC, 60 KCl, 70 KF, 15 NaCl, 5 HEPES, 5 EGTA (mM)) and IonFlux 16-well plates were obtained from Fluxion Bioscences Inc. (South San Francisco, CA, USA). Sodium chloride solution 0.9% was from Grifols Engineering, S.A. (Barcelona, Spain). CVD Milliplex^®^ Map KIT was from Millipore^®^ Iberica S.A. (Billerica, MA, USA). General Health Profile (GHP) chemistry panel was obtained from IDEXX Laboratories (Barcelona, Spain). High sensitivity rat cardiac Troponin-I ELISA kit was from Life diagnostics (Wester Chester, PA, USA).

**Table 2:** Plasmatic levels of biochemical markers in OA-, DTX-1- and YTX-treated rats and controls.

Time		Before	After	Physiologic Range
Biochemical Parameter				
Control (*n* = 9)	BUN (mg/dL)	17 ± 1.0	23.7 ± 1.1 **	20.3–25.5
	CREA (mg/dL)	0.4 ± 0.0	0.4 ± 0.0	0.5–0.92
	PHOS (mg(dL)	6.2 ± 0.2	6.4 ± 0.9	4.2–8.33
	Ca (mg/dL)	10.2 ± 0.1	10.1 ± 0.1	9.6–11.86
	TP (g/dL)	5.2 ± 0.1	3.9 ± 0.1 **	5.00–7.7
	ALB (g/dL)	2.8 ± 0.1	1.9 ± 0.1 **	2.9–4.6
	GLOB (g/dL)	2.4 ± 0.1	2.0 ± 0.1 **	2.1–3.1
	ALT (U/L)	49.1 ± 10.9	52.2 ± 23.8	32.7–84.1
	ALKP (U/L)	130.4 ± 10.1	76.3 ± 6.5 **	82.8–297.3
	CHOL (mg/dL)	59.1 ± 15.6	56 ± 2.2 *	41.1–59.1
	CK (U/L)	131.4 ± 7.4	248.9 ± 28.0 **	494–4132
OA (*n* = 5)	BUN (mg/dL)	18.3 ± 1.5	28.0 ± 0.9 **	20.3–25.5
	CREA (mg/dL)	0.5 ± 0.1	0.6 ± 0.0	0.5–0.92
	PHOS (mg(dL)	6.9 ± 1.0	8.0 ± 0.7	4.2–8.33
	Ca (mg/dL)	10.2 ± 0.1	9.9 ± 0.1 *	9.6–11.86
	TP (g/dL)	5.5 ± 0.2	4.4 ± 0.1 **	5.00–7.7
	ALB (g/dL)	2.8 ± 0.1	1.9 ± 0.1 **	2.9–4.6
	GLOB (g/dL)	2.7 ± 0.1	2.5 ± 0.1	2.1–3.1
	ALT (U/L)	51.5 ± 8.6	95.6 ± 24.6	32.7–84.1
	ALKP (U/L)	110.7 ± 11.8	83 ± 8.3 *	82.8–297.3
	CHOL (mg/dL)	91.3 ± 7.3	69.6 ± 2.9	41.1–59.1
	CK (U/L)	98.5 ± 12.6	251.7 ± 33.3 **	494–4132
DTX-1 (*n* = 5)	BUN (mg/dL)	18 ± 1.1	25.0 ± 2.1 **	20.3–25.5
	CREA (mg/dL)	0.3 ± 0.0	0.3 ± 0.0	0.5–0.92
	PHOS (mg(dL)	7.5 ± 0.4	7.5 ± 0.6	4.2–8.33
	Ca (mg/dL)	10.1 ± 0.1	9.0 ± 0.6	9.6–11.86
	TP (g/dL)	5.5 ± 0.1	4.2 ± 0.1 **	5.00–7.7
	ALB (g/dL)	3.0 ± 0.1	2.0 ± 0.1 **	2.9–4.6
	GLOB (g/dL)	2.5 ± 0.1	2.1 ± 0.1 *	2.1–3.1
	ALT (U/L)	34.5 ± 3.5	21.2 ± 6.2	32.7–84.1
	ALKP (U/L)	123.2 ± 13.7	78.8 ± 8.3 **	82.8–297.3
	CHOL (mg/dL)	74.2 ± 4.2	58.2 ± 8.7	41.1–59.1
	CK (U/L)	113.6 ± 16.6	162.4 ± 26.4	494–4132
YTX (*n* = 7)	BUN (mg/dL)	17.7 ± 1.3	29.8 ± 1.5 **	20.3–25.5
	CREA (mg/dL)	0.5 ± 0.1	0.6 ± 0.2	0.5–0.92
	PHOS (mg(dL)	7.1 ± 1.4	7.0 ± 1.3	4.2–8.33
	Ca (mg/dL)	10.1 ± 0.0	9.5 ± 0.1 **	9.6–11.86
	TP (g/dL)	5.4 ± 0.2	4.1 ± 0.1 **	5.00–7.7
	ALB (g/dL)	2.6 ± 0.1	1.8 ± 0.1 **	2.9–4.6
	GLOB (g/dL)	2.7 ± 0.1	2.5 ± 0.1	2.1–3.1
	ALT (U/L)	23.8 ± 4.5	30.8 ± 8.2	32.7–84.1
	ALKP (U/L)	113.8 ± 8.6	80 ± 6.4 **	82.8–297.3
	CHOL (mg/dL)	82 ± 6.1	64.5 ± 2.9 **	41.1–59.1
	CK (U/L)	151.3 ± 16.7	296 ± 119.0	494–4132

* Statistically significant *versus* levels before administration. ** Statistically significant *versus* levels before administration and at least one of the values out of physiological range.

### 3.2. Cell Line

A Precision^TM^hERG CHO (Chinese hamster ovary) Recombinant cell line (Millipore^®^Iberica S.A., Madrid, Spain) was used for patch clamp experiments. HERG CHO cells were grown in Nut Mix F-12 W/GLUTAMAX-I medium supplemented with 10% FBS and 400 μg/mL of geneticin (complete culture media) in a humidified 5% CO_2_ incubator at 37 °C. They were fed every 24–36 h and passaged every 2–3 days (cell confluence never exceeded 80%). Prior to functional assays, the cell cultures were placed for at least two days at 30 °C in a humidified 5% CO_2_ incubator.

### 3.3. Automated Patch Clamp

The effect of these biotoxins on hERG channel activity was tested using an IonFlux 16 automated patch clamp system (Fluxion Bioscences Inc., South San Francisco, CA, USA). Current measurement experiments were performed in 16-well IonFlux plates that have 8 pairs of cell traps, each trap endowed with 20 trapping sites placed in parallel.

The cells were washed twice with 5 mL of warm DPBS and detached by incubation with 5 mL of warm Detachin^TM^ solution for 10 min at 37 °C. Immediately, 5 mL of warm CHO serum-free culture medium supplemented with 25 mM HEPES was added and the cell suspension was centrifuged at 700 rpm and 19 °C for 5 min. The cell pellet was suspended in CHO serum-free medium supplemented with 25 mM HEPES and centrifuged again in the same conditions. Finally, the cells were washed with EC solution twice and suspended at a concentration of 20 × 10^6^ cells/ml in EC solution for automated patch clamp experiments. Cell viability was always higher than 99% as determined by trypan blue exclusion test.

In order to record hERG currents the voltage protocol applied was as follows: cells were clamped at −80 mV for 100 ms, pulsed to −100 mV for 90 ms and to −50 mV for 50 ms, then depolarized to +20 mV for 5 s and repolarized to −50 mV for 5 s, and finally returned to −80 mV. HERG currents were measured for 300 s after compound application. All experiments were done at 22 °C.

### 3.4. Animals and in vivo Experimental Design

*In vivo* studies were performed with Sprague Dawley female rats of 8–16 weeks of age that weighted between 180 and 260 g. The rats were anesthetized with isoflurane and two catheters were placed in the jugular veins, one for compound administration and sample collection, and the other one for fluid therapy maintenance (5 mL/h). ECG recordings were obtained using lead II. The electrodes were placed on the skin, the negative and positive electrodes were on the right forelimb and on the left hind limb respectively. After surgical manipulation, a period of 15 min lapsed before recording basal ECG in order to ensure stable vital signs. Once the stabilization period was finished, ECG was recorded for 10 min. Then the toxin was administered by intravenous injection. For intravenous injection the solvent of each stock solution (methanol) was evaporated and the toxin was reconstituted in DMSO. Saline solution was added subsequently to provide a final concentration of 8 μg/mL OA, 6.4 μg/mL DTX-1 and 14 μg/mL YTX with a final DMSO concentration of 4% in all solutions. Nine control rats were injected with the same concentration of carrier (DMSO) in saline solution. Blood samples (400 µL) were collected in EDTA tubes right before (control of individual basal levels) and every hour after toxin administration for the detection of cardiac damage biomarkers in plasma. Blood samples (500 µL) were also collected in heparin tubes before toxin administration (control of individual basal levels) and at the end of each experiment for biochemical analysis. All experiments lasted for 240 min after toxin administration, except when the animals died during the experiment. All animal procedures were conducted according to the principles approved by the Institutional Animal Care Committee of the Universidad de Santiago de Compostela.

### 3.5. Electrocardiography

Lead II ECG was recorded at several times during the experiment and every recording period lasted for at least 10 min. An ECG recording was obtained before toxin administration (after the stabilization period) in every experiment and evaluated for alterations. Any abnormality detected in the ECG at this time prompted the interruption of the experiment. After toxin administration, ECG recordings were obtained starting at times 0 min, 50 min, 110 min, 170 min and 230 min. ECG recording speed was 25 mm/s with intervals of 50 mm/s. Heart rate (HR) was evaluated by counting QRS complexes per min. PR interval was measured from P wave onset to QRS complex onset. Duration of QT interval was determined from the onset of QRS complex to the end of T wave. Q waves are often not present in rat ECG, therefore the base of the R wave is used in rodent models as a surrogate for the Q wave [27]. The length and morphology of T waves were evaluated when possible. All intervals were measured at a recording speed of 50 mm/s (Figure 3A). For PR interval, QT interval and T wave length evaluation, six consecutive measurements were done at three non-consecutive, randomly chosen points of every 10 min ECG recording (t-10, t0, t50, t110, t170 and t230). The results are reported for every interval as mean ± standard error of the mean (SEM) of the three randomly selected segments. QT interval was corrected using normalized Bazett’s equation like in previously published studies [36]. The same procedure was followed in control experiments for rats injected with carrier (DMSO) in the absence of toxin.

### 3.6. Cardiac Biomarkers

Cardiac TnI, cTnT and BNP were measured in plasma samples of rats treated with OA, DTX-1 or carrier using a commercial assay based on the Luminex XMap^®^ technology. Cardiac TnI in plasma samples of rats treated with YTX or carrier was determined using a specific ELISA kit. All blood samples collected during *in vivo* experiments were centrifuged immediately after collection to separate the plasma fraction. Plasma samples were stored at −80 °C until their analysis. The commercial Luminex assay is a panel of 3 immunoassays that allows the simultaneous and specific detection of the three rat cardiac biomarkers, cTnI, cTnT and BNP (rat cardiovascular disease panel 1, CVD Milliplex^®^ Map KIT Millipore^®^) in plasma samples (100 µL). The ELISA kit is a high sensitivity assay for the determination of cTnI in plasma samples (50 µL). Both assays were done following the instructions provided by the manufacturers. All samples were assayed in duplicate.

### 3.7. Biochemistry Analysis

Several biochemistry parameters were analyzed in plasma samples collected during *in vivo* experiments and processed in the same way as the plasma samples used for detecting cardiac biomarkers. The analysis was done with the IDEXX VetTest^®^ Chemistry Analyzer. A prepacked panel called General Health Profile (GHP) was used to test a total of 11 parameters: albumin (ALB), alkaline phosphatase (ALKP) alanine aminotransferase (ALT), blood urea nitrogen (BUN), calcium (Ca), cholesterol (CHOL), creatine kinase (CK), creatinine (CREA), globulin (GLOB), phosphorus (PHOS), and total protein (TP).

### 3.8. Data Analysis

Data were plotted as mean ± SEM. Statistical significance was determined by using t test for unpaired data and ANOVA for multiple comparisons. *p* < 0.05 was considered for significance. Sample size was calculated using the following equation: *N* = 2 × [((*Z*_α_ + *Z*_β_)^2^ × σ^2^)/Δ^2^)] where *N* is sample size, σ is the estimated value of standard deviation for population, Δ is the maximum difference between means and *Z*_α_ and *Z*_β_ are constant values which depend on tail number (2), statistical significance level (5%) and potency (90%) of the analysis.

## 4. Conclusions

The evaluation of OA, DTX-1 and YTX cardiotoxicity indicated that none of these toxins exerted acute functional effects on hERG channel activity *in vitro* or on cardiac biomarkers and ECG in rats *in vivo*. The absence of acute functional effects for YTX, in spite of the reported ultrastructural alterations of heart tissue, suggests a short-term compensation. The implications of this ultrastructural damage in heart function for longer periods of exposure to the toxin should be explored because functional compensation may not be sustainable in the long term. Presently, no human reports have been related to cardiac dysfunctions; however, it should be considered that human exposure to the toxin has been limited by the legal regulation that establishes a maximum content of YTX in shellfish destined to human consumption of 1 mg/kg. Hence, these studies are critical for the evaluation of the risks associated to YTX consumption, mostly considering that the regulatory limit for this toxin in seafood has been recently raised from 1 to 3.75 mg of YTX equivalents/kg of shellfish meat, and exposure of humans to the toxin will be increased [48].

## References

[1] EFSA (2008). Opinion of the Scientific Panel on Contaminants in the Food chain on a request from the European Comission on marine biotoxins in shellfish-okadaic acid and analogues. EFSA J..

[2] Valdiglesias V., Prego-Faraldo M.V., Pasaro E., Mendez J., Laffon B. (2013). Okadaic acid: More than a diarrheic toxin. Mar. Drugs.

[3] European Commission (2004). Regulation (EC) No. 853/2004 of the European Parliament and of the Council of 29 April 2004 laying down specific hygiene rules for food animal origin. Off. J. Eur. Union.

[4] Takai A., Bialojan C., Troschka M., Ruegg J.C. (1987). Smooth muscle myosin phosphatase inhibition and force enhancement by black sponge toxin. FEBS Lett..

[5] Honkanen R.E., Codispoti B.A., Tse K., Boynton A.L., Honkanan R.E. (1994). Characterization of natural toxins with inhibitory activity against serine/threonine protein phosphatases. Toxicon.

[6] Tubaro A., Dell’ovo V., Sosa S., Florio C. (2010). Yessotoxins: A toxicological overview. Toxicon.

[7] Terao K., Ito E., Oarada M., Murata M., Yasumoto T. (1990). Histopathological studies on experimental marine toxin poisoning--5. The effects in mice of yessotoxin isolated from Patinopecten yessoensis and of a desulfated derivative. Toxicon.

[8] Aune T., Sorby R., Yasumoto T., Ramstad H., Landsverk T. (2002). Comparison of oral and intraperitoneal toxicity of yessotoxin towards mice. Toxicon.

[9] Tubaro A., Sosa S., Carbonatto M., Altinier G., Vita F., Melato M., Satake M., Yasumoto T. (2003). Oral and intraperitoneal acute toxicity studies of yessotoxin and homoyessotoxins in mice. Toxicon.

[10] Tubaro A., Giangaspero A., Ardizzone M., Soranzo M.R., Vita F., Yasumoto T., Maucher J.M., Ramsdell J.S., Sosa S. (2008). Ultrastructural damage to heart tissue from repeated oral exposure to yessotoxin resolves in 3 months. Toxicon.

[11] De la Rosa L.A., Alfonso A., Vilarino N., Vieytes M.R., Botana L.M. (2001). Modulation of cytosolic calcium levels of human lymphocytes by yessotoxin, a novel marine phycotoxin. Biochem. Pharmacol..

[12] Alfonso A., de la Rosa L., Vieytes M.R., Yasumoto T., Botana L.M. (2003). Yessotoxin, a novel phycotoxin, activates phosphodiesterase activity. Effect of yessotoxin on cAMP levels in human lymphocytes. Biochem. Pharmacol..

[13] Paz B., Daranas A.H., Norte M., Riobo P., Franco J.M., Fernandez J.J. (2008). Yessotoxins, a group of marine polyether toxins: An overview. Mar. Drugs.

[14] Tobio A., Fernandez-Araujo A., Alfonso A., Botana L.M. (2012). Role of yessotoxin in calcium and cAMP-crosstalks in primary and K-562 human lymphocytes: The effect is mediated by anchor kinase A mitochondrial proteins. J. Cell Biochem..

[15] Fernandez-Araujo A., Tobio A., Alfonso A., Botana L.M. (2014). Role of AKAP 149-PKA-PDE4A complex in cell survival and cell differentiation processes. Int. J. Biochem. Cell Biol..

[16] Rubiolo J.A., Lopez-Alonso H., Martinez P., Millan A., Cagide E., Vieytes M.R., Vega F.V., Botana L.M. (2014). Yessotoxin induces ER-stress followed by autophagic cell death in glioma cells mediated by mTOR and BNIP3. Cell Signal.

[17] Tubaro A., Sosa S., Altinier G., Soranzo M.R., Satake M., Della Loggia R., Yasumoto T. (2004). Short-term oral toxicity of homoyessotoxins, yessotoxin and okadaic acid in mice. Toxicon.

[18] Sosa S., Ardizzone M., Beltramo D., Vita F., Dell’Ovo V., Barreras A., Yasumoto T., Tubaro A. (2013). Repeated oral co-exposure to yessotoxin and okadaic acid: A short term toxicity study in mice. Toxicon.

[19] EFSA (2008). Opinion of the Scientific Panel on Contaminants in the Food chain on a request from the European Comission on marine biotoxins in shellfish-yessotoxin group. EFSA J..

[20] EMA (2005). International conference on harmonisation; guidance on s7b nonclinical evaluation of the potential for delayed ventricular repolarization (QT interval prolongation) by human pharmaceuticals; availability. Notice. Fed. Regist..

[21] Guth B.D. (2007). Preclinical cardiovascular risk assessment in modern drug development. Toxicol. Sci..

[22] Stummann T.C., Beilmann M., Duker G., Dumotier B., Fredriksson J.M., Jones R.L., Hasiwa M., Kang Y.J., Mandenius C.F., Meyer T. (2009). Report and recommendations of the workshop of the European centre for the validation of alternative methods for drug-induced cardiotoxicity. Cardiovasc. Toxicol..

[23] Priest B.T., Bell I.M., Garcia M.L. (2008). Role of hERG potassium channel assays in drug development. Channels.

[24] Sanguinetti M.C., Jiang C., Curran M.E., Keating M.T. (1995). A mechanistic link between an inherited and an acquired cardiac arrhythmia: HERG encodes the IKr potassium channel. Cell.

[25] Gintant G.A., Su Z., Martin R.L., Cox B.F. (2006). Utility of hERG assays as surrogate markers of delayed cardiac repolarization and QT safety. Toxicol. Pathol..

[26] Van der Heyden M.A., Smits M.E., Vos M.A. (2008). Drugs and trafficking of ion channels: A new pro-arrhythmic threat on the horizon?. Br. J. Pharmacol..

[27] Farraj A.K., Hazari M.S., Cascio W.E. (2011). The utility of the small rodent electrocardiogram in toxicology. Toxicol. Sci..

[28] Cardinale D., Sandri M.T. (2010). Role of biomarkers in chemotherapy-induced cardiotoxicity. Prog. Cardiovasc. Dis..

[29] Kettenhofen R., Bohlen H. (2008). Preclinical assessment of cardiac toxicity. Drug Discov. Today.

[30] O’Brien P.J. (2008). Cardiac troponin is the most effective translational safety biomarker for myocardial injury in cardiotoxicity. Toxicology.

[31] Garibo D., de la Iglesia P., Diogene J., Campas M. (2013). Inhibition equivalency factors for dinophysistoxin-1 and dinophysistoxin-2 in protein phosphatase assays: Applicability to the analysis of shellfish samples and comparison with LC-MS/MS. J. Agric. Food Chem..

[32] Tubaro A., Sosa S., Bornancin A., Hungerford J., Botana L.M. (2008). Seafood and freshwater toxins. Pharmacology, Physiology and Detection. Pharmacology and Toxicology of Diarrheic Shellfish Toxins.

[33] Katchman A.N., Koerner J., Tosaka T., Woosley R.L., Ebert S.N. (2006). Comparative evaluation of HERG currents and QT intervals following challenge with suspected torsadogenic and nontorsadogenic drugs. J. Pharmacol. Exp. Ther..

[34] Mohammad S., Zhou Z., Gong Q., January C.T. (1997). Blockage of the HERG human cardiac K+ channel by the gastrointestinal prokinetic agent cisapride. Am. J. Physiol..

[35] Ohtani H., Taninaka C., Hanada E., Kotaki H., Sato H., Sawada Y., Iga T. (2000). Comparative pharmacodynamic analysis of Q-T interval prolongation induced by the macrolides clarithromycin, roxithromycin, and azithromycin in rats. Antimicrob. Agents Chemother..

[36] Ferreiro S.F., Vilarino N., Carrera C., Louzao M.C., Santamarina G., Cantalapiedra A.G., Rodriguez L.P., Cifuentes J.M., Vieira A.C., Nicolaou K.C. (2014). *In vivo* arrhythmogenicity of the marine biotoxin azaspiracid-2 in rats. Arch. Toxicol..

[37] Ogino H., Kumagai M., Yasumoto T. (1997). Toxicologic evaluation of yessotoxin. Nat. Toxins.

[38] Moulin M., Piquereau J., Mateo P., Fortin D., Rucker-Martin C., Gressette M., Lefebvre F., Gresikova M., Solgadi A., Veksler V. (2015). Sexual dimorphism of doxorubicin-mediated cardiotoxicity: Potential role of energy metabolism remodeling. Circ. Heart Fail.

[39] Mahmoodzadeh S., Fliegner D., Dworatzek E. (2012). Sex differences in animal models for cardiovascular diseases and the role of estrogen. Handb. Exp. Pharmacol..

[40] Fernandez D.A., Louzao M.C., Fraga M., Vilarino N., Vieytes M.R., Botana L.M. (2014). Experimental basis for the high oral toxicity of dinophysistoxin 1: A comparative study of DSP. Toxins.

[41] Kemp C.D., Conte J.V. (2012). The pathophysiology of heart failure. Cardiovasc. Pathol..

[42] Brady S., York M., Scudamore C., Williams T., Griffiths W., Turton J. (2010). Cardiac troponin I in isoproterenol-induced cardiac injury in the Hanover Wistar rat: Studies on low dose levels and routes of administration. Toxicol. Pathol..

[43] Walker D.B. (2006). Serum chemical biomarkers of cardiac injury for nonclinical safety testing. Toxicol. Pathol..

[44] Ozer J., Ratner M., Shaw M., Bailey W., Schomaker S. (2008). The current state of serum biomarkers of hepatotoxicity. Toxicology.

[45] Berven G., Saetre F., Halvorsen K., Seglen P.O. (2001). Effects of the diarrhetic shellfish toxin, okadaic acid, on cytoskeletal elements, viability and functionality of rat liver and intestinal cells. Toxicon.

[46] Tonomura Y., Tsuchiya N., Torii M., Uehara T. (2010). Evaluation of the usefulness of urinary biomarkers for nephrotoxicity in rats. Toxicology.

[47] Bonventre J.V., Vaidya V.S., Schmouder R., Feig P., Dieterle F. (2010). Next-generation biomarkers for detecting kidney toxicity. Nat. Biotechnol..

[48] Comission regulation (EU) (2013). Amending Annex III to Regulation (EC) No 853/2004 of the European Parliament and of the Council as Regards the Permitted Limits of Yessotoxins in Live Bivalve Molluscs.

